# A Theoretical Perspective on the Photochemistry of Boron–Nitrogen Lewis Adducts

**DOI:** 10.1021/acs.jpca.3c07016

**Published:** 2024-01-18

**Authors:** Emanuele Marsili, Basile F. E. Curchod

**Affiliations:** Centre for Computational Chemistry, School of Chemistry, University of Bristol, Bristol BS8 1TS, U.K.

## Abstract

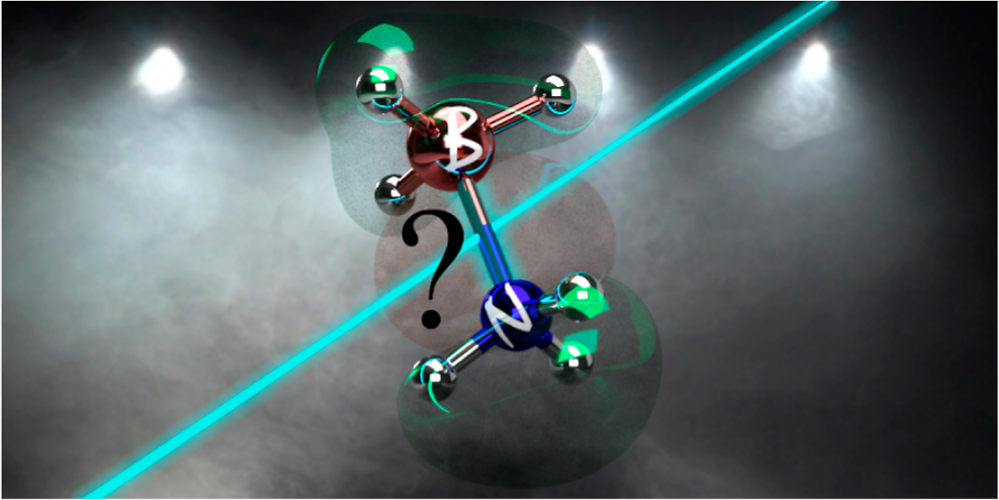

Boron–Nitrogen (B–N) Lewis adducts form a versatile family of compounds with numerous applications in functional molecules. Despite the growing interest in this family of compounds for optoelectronic applications, little is currently known about their photophysics and photochemistry. Even the electronic absorption spectrum of ammonia borane, the textbook example of a B–N Lewis adduct, is unavailable. Given the versatility of the light-induced processes exhibited by these molecules, we propose in this work a detailed theoretical study of the photochemistry and photophysics of simple B–N Lewis adducts. We used advanced techniques in computational photochemistry to identify and characterize the possible photochemical pathways followed by ammonia borane and extended this knowledge to the substituted B–N Lewis adducts pyridine-borane and pyridine-boric acid. The photochemistry observed for this series of molecules allows us to extract qualitative rules to rationalize the light-induced behavior of more complex B–N-containing molecules.

## 1 Introduction

The versatility of Lewis acid–base adducts, characterized by a dative bond between a Lewis acid and Lewis base, has been extensively used for a plethora of molecular applications like organic synthesis,^1,2^ catalysis,^3−5^ and production of new types of dyes for solar cells^6^ or polymers.^7,8^ More recently, a particular boost of interest has emerged for the photochemistry and photophysics of Lewis adducts, with potential applications for functional materials,^7,9,10^ sensors,^11,12^ or optoelectronic devices.^13−15^ For example, the inclusion of a boron atom in polycyclic aromatic hydrocarbons leads to highly stable photoactive molecules, whose optical properties can be altered upon adduct formation with Lewis bases to act as sensors.^13,14^ These B–N Lewis adducts, however, exhibit a rather peculiar photochemical behavior involving excited-state photodissociation and an unexpected double fluorescence. Interestingly, the light-induced dissociation is not unique to this class of Lewis adducts and has been observed for several compounds containing a constrained boron center paired with a relatively weak Lewis base.^10,12,16−18^

Very little is known to date about the photochemistry and photophysics of B–N Lewis adducts. This observation comes as a surprise considering the important body of work on this class of molecules for functional materials and their rather unexpected photochemical properties. Only a few studies tried to connect the strength of the B–N bond to the observed photodissociation behavior.^13,14,17,18^ However, the relation between the strength of the B–N bond in the ground electronic state of the molecule and its potential weakening in the excited states is far from trivial given our general lack of understanding of the electronic-state characters involved in the photochemistry for these molecules.

Perhaps even more surprising is the fact that our unawareness of the photochemistry and photophysics of Lewis adduct extends to ammonia borane, H_3_N–BH_3_, the simplest B–N Lewis adduct. This molecule, often considered as a prototypical model to discuss B–N adducts, has received increased attention in the past years for its connection with hydrogen-storage materials.^19−23^ As a result, the physical properties of H_3_N–BH_3_ were carefully analyzed by several spectroscopic techniques such as microwave,^24^ NMR,^25^ IR,^26^ Raman,^27^ single-photon ionization,^28^ photoelectron spectroscopy,^29,30^ X-ray crystallography,^31^ neutron powder diffraction,^32^ and inelastic neutron scattering.^33^ In addition, numerous computational works focused on obtaining ground-state equilibrium geometries,^34,35^ vibrational frequencies and zero-point energy,^34−36^ as well as its charge transfer characteristics^37^ and ground-state dissociation barrier.^38^ Besides a single theoretical absorption cross-section of H_3_N–BH_3_,^39^ no detailed information about the photochemistry and photophysics of the prototypical ammonia borane appear to exist in the literature, to the best of the authors’ knowledge.

**Figure 1:**
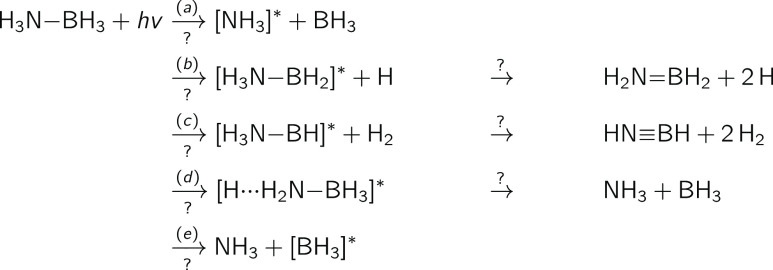
Possible photodissociation channels for H_3_N–BH_3_. [A]* denotes that species A is in an excited electronic state. Question marks are added to stress that these mechanisms are purely speculative; it is the goal of this work to assess whether such processes are possible.

**Figure 2:**
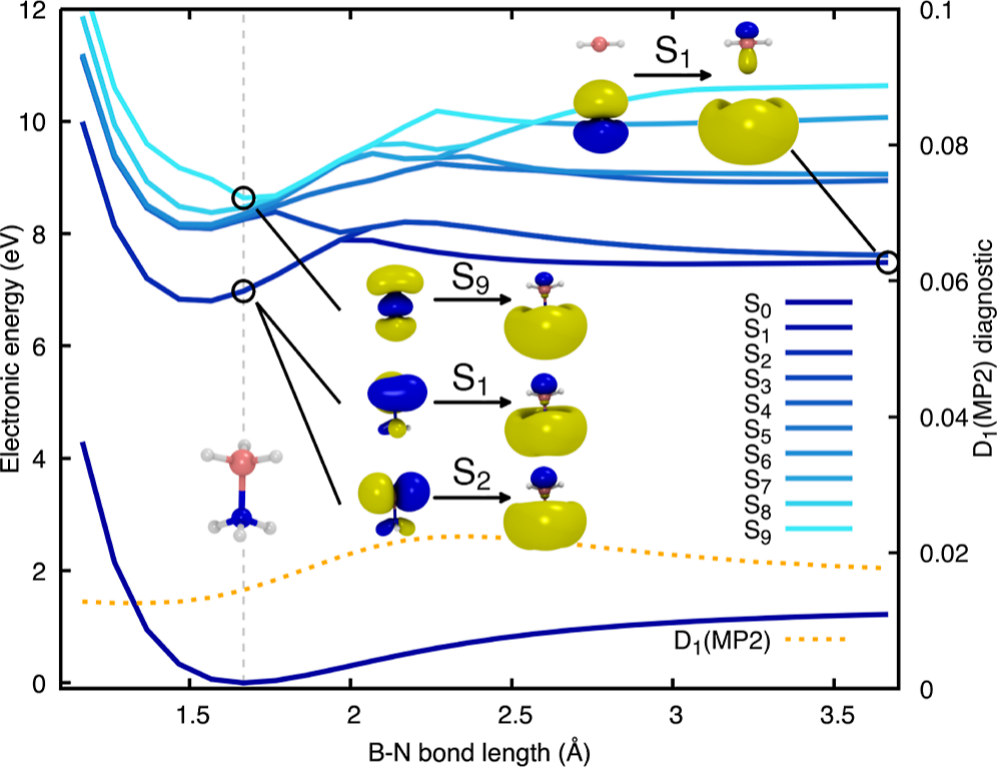
Relaxed scan along the B–N bond length of H_3_N–BH_3_ obtained for the ground electronic state with MP2/aug-cc-pVDZ. Excited electronic energies were obtained with ADC(2)/aug-cc-pVDZ. The FC point is indicated by the gray vertical dashed line with the corresponding geometry as inset. The lowest nine electronic states are depicted in shades of blue: from S_0_ in dark blue to S_9_ in light blue. The D_1_ diagnostic for the MP2/aug-cc-pVDZ ground state is reported as a dotted orange line. The NTOs of the S_1_/S_2_ (E) and S_9_ (A_1_) electronic states at the FC geometry and the S_1_ (A_1_) state at the last point of the relaxed scan are given as insets.

Inspired by the VUV photodissociation of ethane and foreshadowing some of the results presented in this work,^40^ we summarize in Figure 1 the potential photodissociation channels that H_3_N–BH_3_ may encounter. Upon light excitation, ammonia borane can relax to its ground electronic state via internal conversion. The hot ammonia borane formed in the ground electronic state could then possibly dissociate into NH_3_ and BH_3_, with both fragments being in their ground electronic state (Figure 1d). Alternatively, H_3_N–BH_3_ could dissociate in an excited electronic state, yielding NH_3_ or BH_3_, with one of these fragments still being in an excited electronic state (Figure 1a,e). Photoexcitation of H_3_N–BH_3_ could also produce either H_2_ and H_2_N=BH_2_ (Figure 1b) or 2H_2_ and HN≡BH (Figure 1c). The observation of such potential nonradiative deactivation channels is likely to depend on the wavelength of the light source used to photoexcite ammonia borane. One may also wonder how altering the electronic properties of the Lewis base or the Lewis acid forming the adduct could alter the nonradiative deactivation pathways of a B–N adduct. In the following, we will focus on simple alterations of ammonia borane such as pyridine-borane (Py–BH_3_) or pyridine-boric acid (Py–B(OH)_3_).

Hence, the main objectives of this work consist in (i) investigating the excited electronic states of H_3_N–BH_3_, (ii) identifying the possible photodissociation pathways of H_3_N–BH_3_ and their dependence on the excitation wavelength, (iii) extending this knowledge to the more complex B–N Lewis adducts pyridine–BH_3_ (Py–BH_3_) and pyridine–B(OH)_3_ (Py–B(OH)_3_), and (iv) proposing qualitative guidelines to predict the photochemistry of general B–N adducts. To achieve these goals, we propose to investigate theoretically these three B–N Lewis adducts by exploiting a combination of quantum-chemical calculations and excited-state (nonadiabatic) molecular dynamics simulations. These computational techniques give access to potential energy curves describing the possible nonradiative pathways, photoabsorption cross-sections, and wavelength-dependent quantum yields (building upon a protocol developed in our group to study atmospheric volatile organic compounds^41−44^). We start this work by detailing the computational methodologies employed for our calculations (Section 2), before presenting our main results in Section 3. We first focus on the possible photochemical pathways experienced by ammonia borane (Section 3.1), and we offer a benchmark for the level of electronic-structure theory used in this work. We then present our results for the photochemistry of Py–BH_3_ (Section 3.2) and Py–B(OH)_3_ (Section 3.3) and contrast them with the excited-state dynamics of ammonia borane. Finally, we determine a set of qualitative rules for the photochemistry of B–N adducts and used them to interpret experimental findings from the literature (Section 3.4).

## 2 Methods

### 2.1 Electronic Structure and Benchmark of Electronic-Structure Methods for H_3_N–BH_3_

Given the potentially rich photochemistry of B–N Lewis adducts, an adequate choice of electronic-structure methods is critical to correctly capture the different photodissociation pathways. Following an extensive benchmark (see below), we opted for a combination of MP2 (Møller–Plesset perturbation theory up to second order) and ADC(2) (algebraic diagrammatic construction of second order).^45,46^ All calculations were performed with Turbomole 7.4.1,^47^ using frozen cores and the resolution of identity approximation.^48^

Results obtained with MP2 and ADC(2) at the minimum-energy structure in S_0_ were validated against EOM-CCSD (equation-of-motion coupled-cluster singles and doubles, performed with Gaussian09^49^), the multiconfigurational method SA-CASSCF (state-averaged complete active space self-consistent field),^50−52^ and XMS-CASPT2 (extended multistate complete active space second-order perturbation theory).^53,54^ Different basis sets were tested: the correlation-consistent polarized (cc-pVDZ, cc-pVTZ, cc-pVQZ, and the respective augmented sets)^55^ and the Karlsruhe (def2-SVPD) basis sets.^56−58^ BAGEL 1.2 was used for all SA-CASSCF and XMS-CASPT2 calculations.^59^ SA-CASSCF and XMS-CASPT2 calculations were also employed to assess the reliability of ADC(2) along the photodissociation pathways of H_3_N–BH_3_, particularly focusing on the ground electronic state and its potential multireference character. All calculations using BAGEL employed density fitting and frozen cores. For all XMS-CASPT2 calculations, we employed a real vertical shift of 0.5 hartree and the SS-SR contraction scheme.

For the benchmark of the electronic-structure methods, we investigated four potential dissociation channels for H_3_N–BH_3_, namely, (a) the B–N dissociation, (b) the B–H dissociation, (c) the concerted dissociation of H_2_ via two B–H bonds breaking, and (d) the N–H elongation. These processes were studied via a combination of relaxed scans in the ground or first excited electronic state and linear interpolation in internal coordinates (LIICs) connecting critical points of the potential energy surfaces. Relaxed scans in the ground state, used for (a) and (b), and in the first excited state, used for (d), have been performed with MP2/aug-cc-pVDZ and ADC(2)/aug-cc-pVDZ, respectively. LIICs, connecting the Franck–Condon (FC) point (optimized with MP2/aug-cc-pVDZ and showing a staggered configuration), the S_1_ minimum (optimized with ADC(2)/aug-cc-pVDZ), and the  and  minimum-energy conical intersections (MECIs, optimized with XMS-CASPT2/aug-cc-pVDZ), were used to describe the dissociation channels (b) and (c). To benchmark the results obtained from MP2 and ADC(2), we obtained XMS-CASPT2/aug-cc-pVDZ energies along these relaxed scans and LIICs, using two different combinations of active spaces and state averaging. The first combination, used for pathways (a), used a state averaging over 13 singlet states and an active space composed of 8 electrons in 9 orbitals (see Figure S1 for a representation of the SA(13)-CASSCF(8/9) natural orbitals and their labeling). The second combination, used for pathways (b), (c), and (d), employed 5 singlet states for the state averaging and an active space of 4 electrons in 8 orbitals (see Figure S2 for a representation of the SA(5)-CASSCF(4/8) natural orbitals and their labeling).

### 2.2 Ground-State Sampling and Photoabsorption Cross-Sections

For each molecule studied—ammonia borane, pyridine-borane, and pyridine-boric acid—a set of 500 initial conditions were sampled from an ab initio molecular dynamics run using MP2/aug-cc-pVDZ for the electronic-structure theory and a quantum thermostat (QT), performed with the ABIN code^60^ coupled to the Turbomole 7.4.1^47^ Parameters for the quantum thermostat were taken from the GLE4MD webpage,^61^ using a target temperature *T* = 296 K and the following parameters: Ns = 6, *ℏ*ω/*kT* = 20, strong coupling. The time step for the ab initio molecular dynamics was 20 atomic time units (atu). The equilibration time was determined by monitoring the convergence of the average kinetic energy temperature. The nuclear configurations, used subsequently as initial conditions with the corresponding nuclear velocities, were sampled each 2000 atu during the production run. We note that the rotamers of H_3_N–BH_3_ (along the B–N bond) were adequately sampled thanks to the QT dynamics.

The initial conditions were used to calculate the photoabsorption cross-sections of H_3_N–BH_3_, Py–BH_3_, and Py–B(OH)_3_ at the ADC(2)/aug-cc-pVDZ and ADC(2)/cc-pVDZ level of theory. None of the molecules studied contain a carbonyl group, which leads to artificial crossings between the first and the ground electronic states with ADC(2)/MP2.^62^ An augmented basis set is needed to describe the Rydberg character of the H_3_N–BH_3_ excited states properly. In contrast, the use of an augmented basis is less important for the Py–BH_3_ and Py–B(OH)_3_ adducts, characterized by only low-lying valence excited states (see further discussion below). All spectral transitions were broadened with Lorentzians using a phenomenological broadening of 0.05 eV. The resulting photoabsorption cross-sections for H_3_N–BH_3_ and Py–BH_3_ were obtained by averaging the contribution of all 500 geometries using the nuclear ensemble approach (NEA).^63^ Due to the weak B–N bond of the Py–B(OH)_3_ adduct, 97 out of the 500 initial conditions sampled from the QT dynamics had a B–N bond longer than 2.1 Å. To avoid any bias in our result, we calculated the photoabsorption cross-section employing the 403 initial conditions exhibiting a B–N bond shorter than 2.1 Å (see Figure S3 for the B–N distribution and Figure S4 for the photoabsorption cross-section). The NEA and the spectrum were calculated with Newton-X version 2.4.^64^

### 2.3 Excited-State Dynamics and Quantum Yields

The excited-state (nonadiabatic) molecular dynamics simulations were performed with the trajectory surface hopping (TSH) algorithm.^65^ TSH dynamics were carried out using ADC(2)/aug-cc-pVDZ for H_3_N–BH_3_ and ADC(2)/cc-pVDZ for Py–BH_3_ and Py–B(OH)_3_, with a time step of 0.5 fs and using Newton-X version 2.4 coupled with Turbomole.^66^ The nonadiabatic couplings were obtained using the overlap-based time-derivative couplings computed using the orbital derivative scheme,^67^ and the kinetic energy was adjusted by rescaling the nuclear velocity vector isotropically following a successful hop. The electronic populations were corrected to prevent overcoherence using the energy-based decoherence correction of Granucci and Persico.^68^

The TSH dynamics were started from the initial conditions used for the NEA (see above) and grouped into different energy windows based on the calculated photoabsorption cross-sections of H_3_N–BH_3_, Py–BH_3_, and Py–B(OH)_3_. Three energy windows with a width of 1.0 eV were selected for H_3_N–BH_3_ and Py–BH_3_. For H_3_N–BH_3_, the windows were centered at 6.0, 7.0, and 8.0 eV, while for Py–BH_3_ the three windows were centered at 4.0, 5.0, and 6.0 eV. Two windows instead were selected for the low-energy band of Py–B(OH)_3_, centered at 4.75 and 5.25 eV, each with a width of 0.5 eV. The choice of the width is somewhat arbitrary and basically guided by the balance between computational cost and the interplay between excited states observed from the photoabsorption cross-sections. The initial conditions were selected randomly in each window (i.e., not weighted by their individual oscillator strength), and their statistics are reported in Table S1. For Py–B(OH)_3_, we only used initial conditions with a B–N bond shorter than 2.1 Å. All TSH trajectories were stopped when the S_0_/S_1_ energy gap became smaller than 0.05 eV, and the last point of the dynamics is used to assess the type of nonradiative process suffered by the molecule; we called this analysis the “fraction of trajectories per CI” in the following. The standard deviations of the fraction of trajectories per CI (or photodissociation channel) were estimated following the method of Persico and Granucci.^69^

## 3 Results and Discussion

### 3.1 Photochemistry of Ammonia Borane

In the following, we focus our attention on the different deactivation pathways suffered by ammonia borane upon photoexcitation in its lowest excited electronic states. We first discuss the electronic character of the low-lying singlet states of H_3_N–BH_3_ in the FC region, before investigating the behavior of its electronic energies along a stretch of the B–N bond. Inspired by the VUV photodissociation of ethane,^40^ which is structurally and electronically similar to H_3_N–BH_3_, we then investigate the potential H and H_2_ photodissociation channels of ammonia borane. In contrast with ethane,^40^ the introduction of the B and N atoms in ammonia borane produces a strong asymmetry in the electronic structure of the excited electronic state, leading to a possible different behavior of the molecule when H photodissociation is triggered from the BH_3_ or the NH_3_ moiety.

The low-lying excited electronic states of H_3_N–BH_3_ are characterized by a strong Rydberg character, which imposes some constraints on the theoretical methods that can be used for their description. For example, it is well established that LR-TDDFT, within its practical approximations, usually yields a poor description of Rydberg states.^70^ In addition, the high density of electronic states in the UV-VUV regime challenges the use of multireference methods based on a state-averaging procedure, in particular, for excited-state dynamics. In this context, ADC(2) appears to be a viable alternative, given its compromise between efficiency and accuracy. In the following sections, we propose a discussion of the electronic states of ammonia borane and its possible photodissociation channels combined with a careful benchmark of ADC(2) to assess its validity along the different decay pathways of this molecule.

#### 3.1.1 Vertical Excitation Energies of Ammonia Borane

We first compare the excitation energies obtained with ADC(2), EOM-CCSD, and XMS-CASPT2 (using different basis sets) to confirm that ADC(2) can provide an adequate description of the Rydberg excited electronic states of H_3_N–BH_3_. The combination of ADC(2) with the aug-cc-pVDZ basis set yields vertical transition energies and oscillator strengths in excellent agreement with EOM-CCSD/aug-cc-pVQZ for the nine lowest excited states investigated (Table S2). These results are further validated by XMS-CASPT2 calculations, even though some oscillator strengths appear weaker with this method—a possible weakness of XMS-CASPT2 already highlighted in earlier work (see, for example, ref (71)). Based on this first validation of ADC(2)/aug-cc-pVDZ in the FC region, we now extend our benchmark to the investigation of the photodissociation pathways expected for H_3_N–BH_3_. All calculations presented from this point on will use an aug-cc-pVDZ basis set, except if stated otherwise.

#### 3.1.2 Pathway (a): Photodissociation of the B–N Bond in Ammonia Borane

Let us first focus on the dissociation of the B–N bond. In the ground electronic state, the B–N dissociation follows a heterolytic cleavage such that the Lewis acid and base are released as neutral species. But how does such a dissociation take place in the excited electronic states?

To answer this question, we performed a relaxed scan along the B–N bond length at the MP2 level of theory in the ground electronic state and used the obtained geometries to calculate the electronic energies for the different excited electronic states of interest with ADC(2). The resulting electronic energies for the first nine electronic states are represented in Figure 2.

At the FC point ground–state-optimized geometry, indicated by the gray dashed line in Figure 2—the two degenerate lowest electronic states (E symmetry) have a σ_BH_ → Ryd_3s_ character. The σ_BH_ is a linear combination of the σ orbitals constituting the B–H bonds, while Ryd_3s_ is the Rydberg-like 3s virtual orbital on the nitrogen. The other higher excited states have a similar nature and are all characterized by the same donating orbital (σ_BH_). However, the accepting orbital is either a Rydberg-like 3ps orbital of the nitrogen (Ryd_3p_) or a more complex orbital involving the 3s orbital on the boron (see Figure S1). The trend changes when looking at the ninth electronic state (A_1_ symmetry), which, at the FC point, corresponds to a n_N_ → Ryd_3s_ transition, where n_N_ is the lone pair of the nitrogen.

Now that electronic-state characters are defined at the FC point, let us follow the evolution of the electronic states along the B–N coordinate. Upon elongation of this bond, we notice a strong destabilization of the σ_BH_ → Ryd_3s_ states, in stark contrast with the n_N_ → Ryd_3s_ state that becomes the first excited state for a B–N bond longer than 2 Å. This change of electronic character between adiabatic electronic states is validated by monitoring the NTOs for the first excited state at the last point of the relaxed scan (see the inset in Figure 2 at ∼3.6 Å), which confirms the local excitation of ammonia from its lone pair to the Ryd_3s_ orbital. This conclusion is further corroborated by noting that the energy gap is 6.26 eV between this excited electronic state and the ground state at the last point of the LIIC, a value consistent with the first excited state of NH_3_, calculated at 6.20 eV with the same level of electronic-structure theory.

From a benchmark perspective, the D_1_ diagnostic for the MP2 ground state remains well below the recommended limit value of 0.04^72^ along the entire B–N relaxed scan (dotted orange line in Figure 2). This observation suggests that the ground state is still well described by a (closed-shell) single configuration, consistent with the suggested heterolytic dissociation. We also compared the electronic energies obtained with ADC(2) to those calculated with SA(13)-CASSCF(8/9) and XMS(13)-CASPT2(8/9) on the support of the relaxed scan (Figure S5). The three different methods offer similar trends for the behavior of the electronic states along the B–N dissociation coordinate, and we highlight here the rather good agreement between ADC(2) and XMS-CASPT2 (despite XMS-CASPT2 not describing all the possible Rydberg states due to the limited active space employed).

The presence of a n_N_ → Ryd_3s_ electronic state, leading to the weakening of the B–N bond by removing an electron from the nitrogen lone pair, could offer an explanation for the photodissociation process observed in different experimental studies.^13,14,17,18^ If the ammonia borane molecule is photoexcited in its lowest E states (S_1_ and S_2_ in the FC region), an elongation of B–N could result in an adiabatic transfer to an n_N_ → Ryd_3s_ character occurring at ∼2 Å (see Figure 2), even if this process would require to overcome an activation barrier. Once the electronic character of the molecule evolved to that of the dissociative state, H_3_N–BH_3_ should spontaneously dissociate, resulting in the formation of an electronically excited ammonia molecule and a borane in its ground state (see S_1_ inset at long B–N distance in Figure 2), in agreement with the reaction channel (a). We note that pathway (e), leading to the formation of an excited borane and ground-state ammonia, is not observed in the range of electronic energies studied here.

#### 3.1.3 Pathway (b): H Photodissociation from the BH_3_ Moiety

We investigate here the H photodissociation occurring from the BH_3_ moiety, which we expect to be more favorable given the character of the first excited electronic state discussed above. We note that, in contrast with the B–N stretch studied in the previous section, the B–H and N–H stretching modes are not totally symmetric and lift the degeneracy of the E states. As a result, we do not use labels with spatial symmetry in the following.

**Figure 3:**
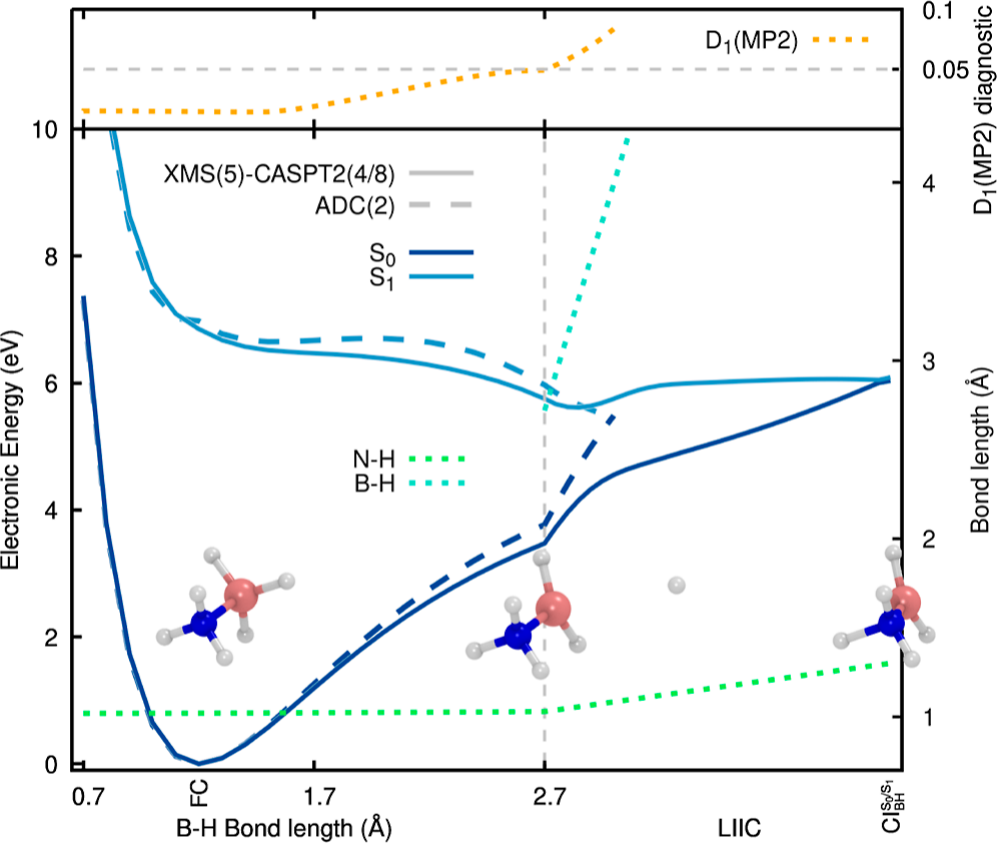
Electronic-energy profile along the H photodissociation of ammonia borane from the BH_3_ moiety. The profile is composed of a relaxed scan along the H dissociation coordinate (performed in the ground electronic state with MP2/aug-cc-pVDZ), combined with a LIIC that connects the last point of the relaxed scan to the geometry of the . The separation between the scan and the LIIC is indicated by a vertical gray line. The two lowest electronic states are colored dark and light blue, respectively. ADC(2)/aug-cc-pVDZ and XMS(5)-CASPT2(4/8)/aug-cc-pVDZ profiles are highlighted by dashed and solid lines, respectively. The relevant B–H and N–H bond lengths are shown as blue-green and green dotted lines along the profile. The D_1_ diagnostic for the MP2/aug-cc-pVDZ ground state is reported as a dotted orange line in the upper panel. In the inset, we report three critical structures, namely, the FC geometry, an intermediate geometry (the connection between the relaxed scan and the LIIC), and the  geometry. In the latter molecular representation, the leaving H atom is too far from the NH_3_–BH_2_ molecule to be visible.

The B–H bond cleavage is illustrated in Figure 3, where a relaxed scan (for the ground state, MP2) is combined with a LIIC toward the optimized geometry for the B–H dissociation . The first excited electronic state (S_1_) is stabilized upon B–H elongation, in agreement with its σ_BH_ → Ryd_3s_ character (see NTOs in Figure 2), and a B–H dissociation can proceed with almost no activation barrier, suggesting a fast photodissociation of the H atom from the BH_3_ moiety (Figure 3). Up to the last point of the scan (indicated with a dashed vertical gray line in Figure 3), the MP2/ADC(2) and XMS-CASPT2 electronic energies are in excellent agreement. At the last point of the scan, the B–H length is 2.7 Å (the sum of the van der Waals radii of H and B is ∼3.0 Å), while the S_0_/S_1_ energy gap is still >2 eV. This observation suggests that the B–H bond is fully broken in the excited electronic state, and the B–H elongation may not be the only coordinate that brings the molecule to the intersection region.

How does the molecule reach the S_1_/S_0_ intersection after the photodissociation of H from the BH_3_ moiety? The answer can be found by inspecting the LIIC connecting the relaxed scan to the  (Figure 3, right side of the vertical dashed gray line). The  geometry is characterized by NH_3_–BH_2_ and an H atom completely dissociated (B–H distance is ∼9 Å). The NH_3_–BH_2_ molecule exhibits a longer N–H bond (longer by ∼0.3 Å with respect to the last point of the relaxed scan, see green dotted line in Figure 3). This distortion of the NH_3_–BH_2_ moiety appears to take the molecule toward the  once the B–H bond is cleaved in the first excited electronic state. The N–H elongation process is somewhat captured by ADC(2), which shows a point of degeneracy between the S_1_ and S_0_ electronic energies. Still, care needs to be taken, as the S_1_/S_0_ crossing point occurs at a much shorter N–H bond length when one compares the ADC(2) profile with that obtained with XMS-CASPT2. This fast interception of the S_1_ state by S_0_ is caused by an artificial destabilization of the MP2 ground state, which is clearly visible from the LIIC in Figure 3. This conclusion is reinforced by the sharp increase in the D_1_ diagnostic at the beginning of the LIIC, suggesting that the ground electronic state cannot be described adequately by a single closed-shell configuration. Hence, while the B–H photodissociation of ammonia borane is well described by ADC(2), the subsequent geometrical distortions leading to the S_1_/S_0_ intersection region via the N–H elongation push this method beyond its limits.

It is worth noting that the N–H elongation discussed above does not necessarily imply that the N–H bond will dissociate but may simply act as a distortion mode to access the intersection region. We will discuss later a different photodissociation channel involving an S_1_/S_0_ crossing mediated by an N–H dissociation that occurs readily after the B–H dissociation. This process follows a similar route to what is depicted in Figure 3 but would likely lead to the release of H_2_ and H_2_N=BH_2_ (as observed in the photodissociation of ethane^40^).

#### 3.1.4 Pathway (c): H_2_ Dissociation from the BH_3_ Moiety

**Figure 4:**
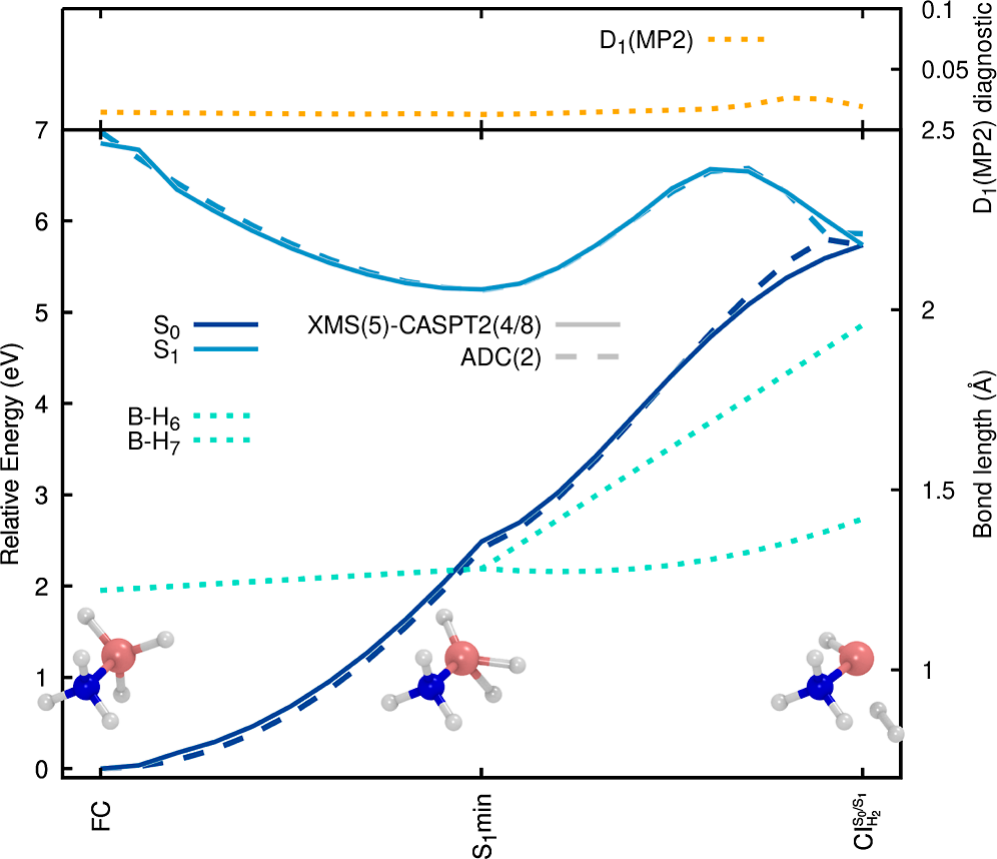
LIIC between the FC, S_1_min, and  geometries of ammonia borane. The two lowest electronic states are depicted in dark and light blue. ADC(2)/aug-cc-pVDZ and XMS(5)-CASPT2(4/8)/aug-cc-pVDZ electronic energies are reported as dashed and solid lines, respectively. The two relevant B–H bonds are shown as blue-green dotted lines. The D_1_ diagnostic for the MP2 ground state is reported as a dotted orange line in the upper panel. In the inset, we provide the three critical structures used to create the LIIC, namely, the FC point, the S_1_min, and the .

The release of H_2_ from photoexcited ammonia borane can occur via the simultaneous dissociation of two H atoms from the BH_3_ moiety. To investigate this process, we perform a LIIC between the FC point optimized with MP2, the S_1_min optimized with ADC(2), and the MECI for the H_2_ release . The electronic energies calculated with MP2/ADC(2) and XMS(5)-CASPT2(4/8) along the LIIC are shown in Figure 4.

The first part of the LIIC connecting the FC point to the S_1_min is characterized by a Jahn–Teller distortion of the molecule along which two out of the three B–H bonds elongate and distort. Such an elongation is visible from the B–H_6_ and B–H_7_ bond lengths displayed as dotted blue-green lines in Figure 4, while the distortion is visible in the S_1_min structure given as an inset. The S_1_ electronic state is stabilized by ∼1.7 eV along this first segment of the LIIC.

The second part of the LIIC—connecting the S_1_min to the —highlights the concerted photodissociation of the two B–H bonds and the release of H_2_, leaving NH_3_–BH. The  geometry indicates that the H–H bond is already formed when the S_0_/S_1_ electronic states cross. While one of the B–H distances is still shorter than 1.5 Å at the  geometry, the interaction between the boron atom and the H_2_ molecule is presumably weak enough to allow the departure of the hydrogen molecule after a return to S_0_. Upon inspection of the S_1_ energy along the LIIC, we can expect that the photodissociation of ammonia borane into H_2_ and NH_3_–BH is energetically feasible, given that the energy barrier along the LIIC is lower than the S_1_ energy at the FC point. We note here that a LIIC does not provide a minimum-energy path, and as such, energy barriers along a LIIC are likely to be overestimated.

Comparing the ADC(2) and XMS-CASPT2 electronic energies along this LIIC reveals excellent agreement between the two methods. This accuracy is also retained close to the CI region where the D_1_ diagnostic remains surprisingly low. Hence, ADC(2) is expected to adequately describe the excited-state dynamics leading to the H_2_ release via the concerted breaking of two B–H bonds.

#### 3.1.5 Pathway (d): H Dissociation from the NH_3_ Moiety

We finally note that electronic energies provided by ADC(2) agree well with those obtained with XMS-CASPT2 for the direct N–H bond breaking upon photoexcitation. To evaluate the likelihood of this direct N–H photodissociation, we performed a relaxed scan in the S_1_ excited electronic state (with ADC(2)) along the N–H bond of H_3_N–BH_3_ (see Figure S6). The energy gained during the relaxation following photoexcitation from the FC point to the S_1_min (see Figure 4) makes a direct N–H photodissociation plausible.

#### 3.1.6 Photoabsorption Cross-Section and Nonadiabatic Dynamics of Ammonia Borane

The main summary of the previous section is that all the proposed photodissociation channels could be accessible upon photoexcitation of ammonia borane, with the likelihood of reaching a given decay pathway being dictated by the initial excitation wavelength and subsequent coupled electron–nuclear dynamics between the excited electronic states. Hence, we now turn our attention to the determination of a photoabsorption cross-section for ammonia borane and the investigation of the potential photochemical pathways triggered at different excitation wavelengths by using nonadiabatic molecular dynamics simulations. Based on the overall good agreement observed between ADC(2) and XMS-CASPT2 for the different photodissociation pathways (and keeping in mind the potential limitations of the former method), we decided to use the ADC(2)/aug-cc-pVDZ level of electronic structure theory for the photoabsorption cross-section of H_3_N–BH_3_ and its nonadiabatic molecular dynamics with TSH (see Section 2 for additional details).

**Figure 5:**
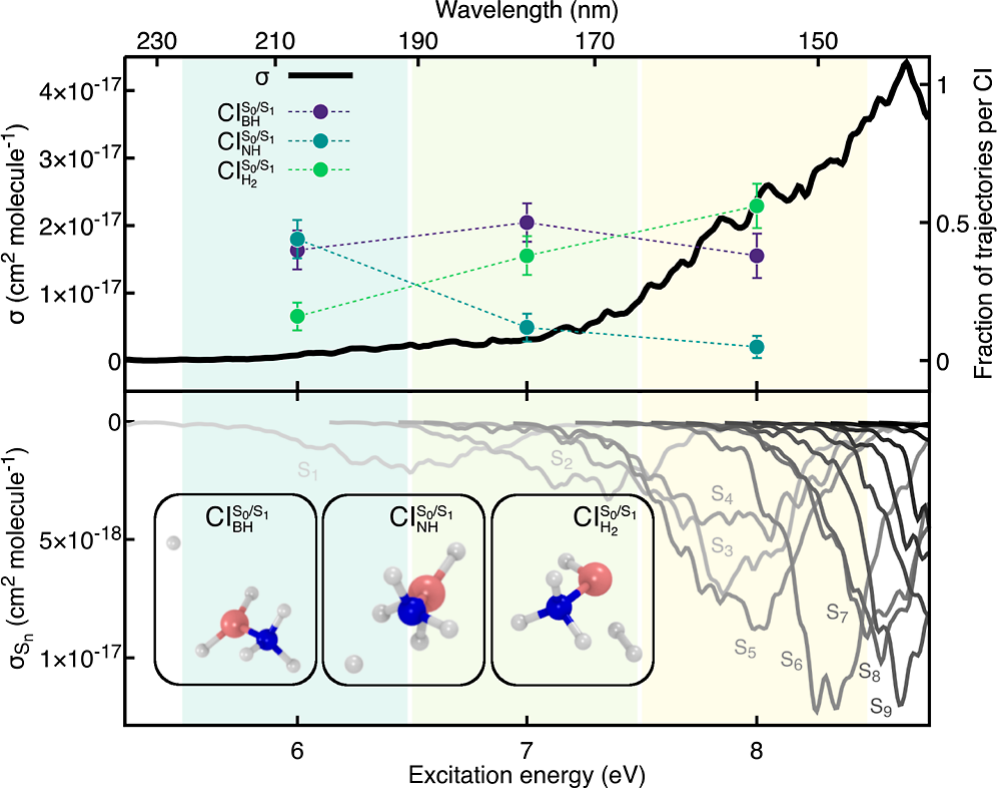
Photoabsorption cross-section and fractions of trajectories reaching a certain CI for H_3_N–BH_3_. Upper panel: full photoabsorption cross-section (black solid line). The spectral range is partitioned into three energy windows, shown as green, light green, and yellow areas. The fraction of trajectories undergoing B–H dissociation (, purple), N–H dissociation (, blue-green), and H_2_ release from the cleavage of two B–H bonds (, light green) is determined for each energy window and reported with filled circles. Lower panel: individual excited-state contributions to the full photoabsorption cross-section depicted with solid lines colored from light gray (S_0_ → S_1_) to black (S_0_ → S_15_). Exemplary molecular structures from the nonadiabatic molecular dynamics, representative of the CI decay for each of the three photodissociation pathways, are shown as insets. ADC(2)/aug-cc-pVDZ is used for the electronic structure.

The total photoabsorption cross-section of H_3_N–BH_3_ is shown in the upper panel of Figure 5 (black solid line) and further decomposed into its different electronic-state contributions (S_0_ → S_*n*_) in the lower panel of the same figure. The first electronic transition (S_0_ → S_1_) spans a rather broad energy range (6–7 eV) and, together with a small contribution from S_0_ → S_2_ excitation, is the main transition present in the first selected energy window (shaded green area) for the subsequent excited-state dynamics simulations. The second and third energy windows (light-green and yellow areas) are more congested and contain electronic excitations to several different electronic states. The intensity of the photoabsorption cross-section grows across the three windows, with S_0_ → S_6_ and S_0_ → S_9_ being the most intense bands in this energy range.

Having defined different regions of interest in the photoabsorption cross-section of ammonia borane, we can now proceed with nonadiabatic dynamics by initiating a swarm of TSH trajectories in each of the three energy windows (green, light green, and yellow areas in Figure 5). Around 50 TSH trajectories were launched in each energy window (see Table S1 for the precise values). Our earlier analysis showed that the fraction of trajectories reaching a particular CI can be associated with a specific photodissociation pathway (upper panel of Figure 5). Three of the four expected channels discussed above were observed in the TSH dynamics: the B–H dissociation , N–H elongation , and the H_2_ release from the concerted cleavage of two B–H bonds . Comparing the fraction of trajectories reaching each of these three CIs shows that the B–H dissociation pathway appears to be largely independent of the excitation energy. In contrast, the concerted cleavage of two B–H bonds becomes more favorable at higher excitation energies, at the expense of the nonradiative decay via the N–H elongation. No direct B–N bond photodissociation in the excited electronic states was observed in the TSH dynamics.

Since we showed that ADC(2) behaves properly when elongating the N–H bond close to the CIs region (see Figure S6), we restarted five trajectories in S_0_ after they reached the  and carried on 500 fs of adiabatic dynamics at the MP2/aug-cc-pVDZ level of theory. We observed a fast reformation of the N–H bond in S_0_ for all five trajectories, followed by an unexpected B–N dissociation in S_0_ due to a vibrationally hot H_3_N–BH_3_ adduct. This B–N dissociation should not be confused with the photodissociation described in Section 3.1.2 where the B–N bond breaking takes place in an excited electronic state and leads to the formation of an electronically excited NH_3_.

Despite the surprisingly rich photochemistry of H_3_N–BH_3_, the photodissociation of the adducts still remains elusive. Given the nature of the low-lying electronic states in H_3_N–BH_3_, one may be tempted to play with substitutions on the NH_3_ or BH_3_ moiety to alter the ordering of the excited electronic states and potentially favor the B–N bond rupture.

### 3.2 Photochemistry of Pyridine Borane

Let us now investigate the photochemistry of pyridine borane, Py–BH_3_. The overall motivations behind the modifications of the Lewis base (and later the Lewis acid) forming the B–N bond are to (i) lower the transition energies to make them more readily accessible for future spectroscopic studies and (ii) favor the excited photodissociation associated with channel (a) from Figure 1, that is, the rupture in an excited electronic state of the B–N bond. From a computational perspective, we will use MP2 and ADC(2) for all calculations given their good performance for ammonia borane and the increase in computational cost resulting from the substitution of NH_3_ by pyridine.

The B–N bond length of Py–BH_3_ is slightly shorter than that of H_3_N–BH_3_ at the optimized ground-state geometry: 1.64 Å vs 1.66 Å, respectively (MP2/aug-cc-pVDZ, see Table S4). This slight shortening of the B–N bond, connected to the sp^2^ hybridization of the nitrogen in pyridine, confirms that the interaction between the Lewis acid and base remains significant. This finding is further corroborated by gas-phase binding energies reported by Potter et al.^73^

#### 3.2.1 Photodissociation of the B–N Bond in Pyridine Borane

**Figure 6:**
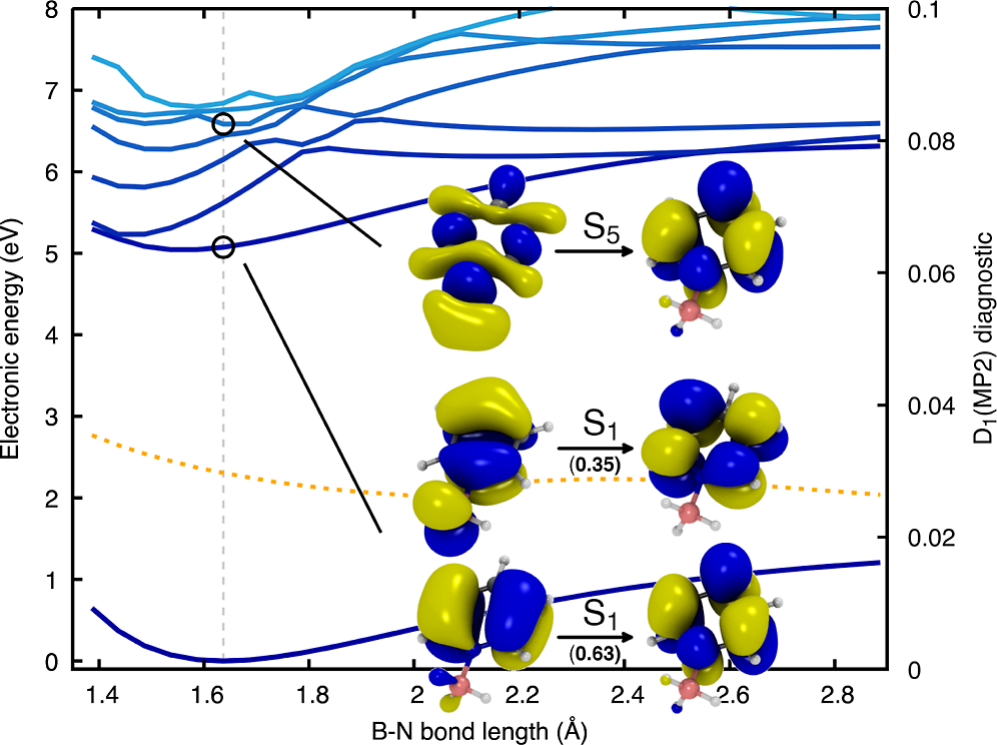
Relaxed scan along the B–N bond length of Py–BH_3_ obtained for the ground electronic state with MP2. Excited electronic energies were obtained with ADC(2)/aug-cc-pVDZ. The FC point is indicated by the gray vertical dashed line. The lowest eight electronic states are calculated along the scan and depicted by lines in shades of blue–from S_0_ in dark blue to S_7_ in light blue. The D_1_ diagnostic for the MP2 ground state is reported as a dotted orange line. NTOs for the S_1_ (two pairs of NTOs are required to describe the first excited state) and S_5_ electronic states at the FC geometry are given as insets.

As for H_3_N–BH_3_, we performed a relaxed scan along the B–N bond with MP2 and used these structures to calculate electronic energies with ADC(2) (see Figure 6). Focusing our attention on the FC geometry, we notice that the first excited state—located at 5 eV above S_0_—can be described by two pairs of NTOs with the following character: π → π* (lower NTO pair with a singular value equal to 0.63) with a small contribution of the type π′ + σ_BH_ → π′* (upper NTO pair with a singular value equal to 0.35). The σ_BH_ contribution to the S_1_ state is similar to the contribution observed for the first two excited states of H_3_N–BH_3_ (in the FC region). However, the first excited state of Py–BH_3_ is 2 eV lower than the first excited state of H_3_N–BH_3_ thanks to the π system of Py, meaning that Rydberg-like orbitals do not contribute to the low-lying electronic states of Py–BH_3_.

Looking at the NTOs describing the S_5_ electronic state of Py–BH_3_ (inset in Figure 6), we notice that the donating orbital exhibits a clear signature of the pyridine lone pair (n_N_). Similar to what was observed for H_3_N–BH_3_, the character of this dissociative electronic state can be followed diabatically from S_5_ in the FC region down to S_1_ for longer B–N distances.

#### 3.2.2 Photoabsorption Cross-Section and Nonadiabatic Dynamics of Pyridine Borane

**Figure 7:**
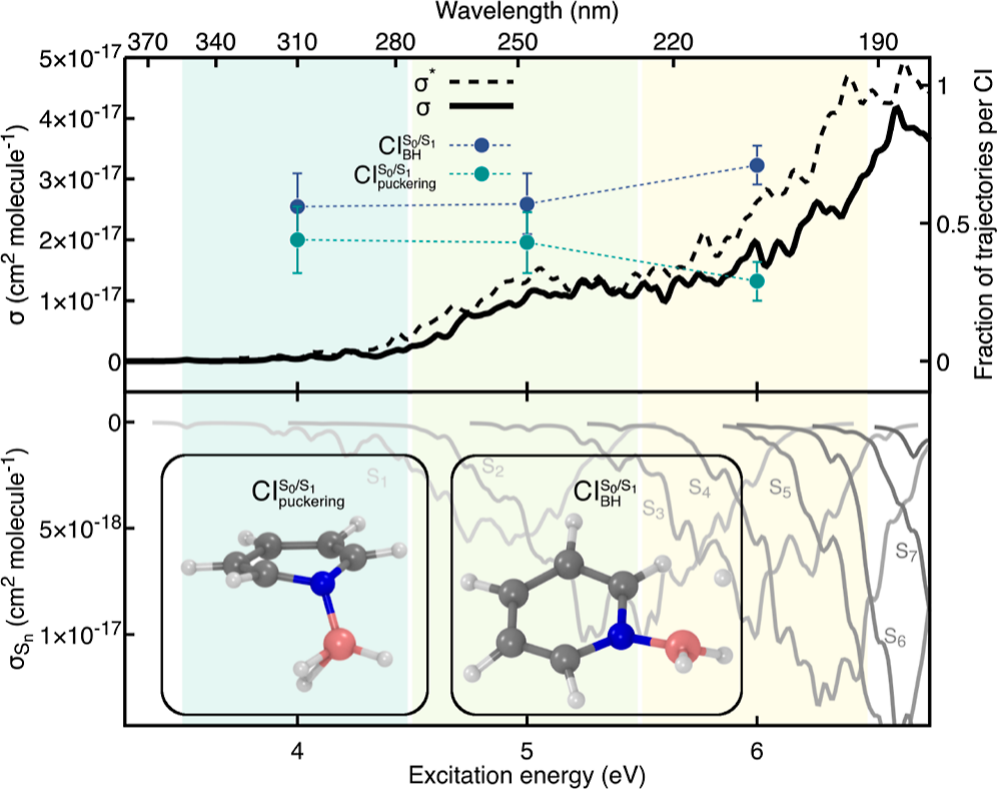
Photoabsorption cross-section and fractions of trajectories reaching a certain CI for Py–BH_3_. Upper panel: full photoabsorption cross-section obtained with ADC(2)/cc-pVDZ (solid black line) and ADC(2)/aug-cc-pVDZ (dashed black line). The spectral range is partitioned into three energy windows shown as green, light green, and yellow areas. The fraction of trajectories undergoing B–H dissociation (, purple) or ring puckering dissociation (, blue-green) is determined for each energy window and reported with filled circles. Lower panel: individual excited-state contributions to the full photoabsorption cross-section depicted with solid lines colored from light gray (S_0_ → S_1_) to dark gray (S_0_ → S_8_). Exemplary molecular structures from the nonadiabatic molecular dynamics, representative of the CI decay for each of the two photodissociation pathways, are shown as insets.

Mirroring Section 5 on ammonia borane, we now discuss the photoabsorption cross-section and nonadiabatic dynamics of pyridine borane. Before doing so, we start with a technical note on the basis set used for the upcoming calculations. Given the reduced presence of Rydberg states in the low-lying excited states of this Lewis adduct, we used the smaller basis set cc-pVDZ instead of aug-cc-pVDZ. Vertical transition energies obtained with ADC(2)/cc-pVDZ are in good agreement with those calculated with ADC(2)/aug-cc-pVDZ (Table S3 in the ESI). To further confirm the validity of removing the diffuse functions, we calculated the photoabsorption cross-section using ADC(2)/cc-pVDZ and ADC(2)/aug-cc-pVDZ (solid and dashed black lines in the top panel of Figure 7). Once again, the two cross-sections show a good overlap, particularly for excitation energies below 6.5 eV. The ADC(2)/aug-cc-pVDZ appears to be only slightly red-shifted with respect to the ADC(2)/cc-pVDZ spectra.

The first energy window (green area in Figure 7) contains only the weak low-energy tail coming from the first excited state with excitation energies extending up to 4.5 eV. The second and third windows (light-green and yellow areas in Figure 7, respectively) cover more electronic transitions–the second window, in particular, contains transitions toward S_1_, S_2_, and S_3_.

The nonadiabatic dynamics simulation of Py–BH_3_ reveals two dominant nonradiative pathways. The first one involves the cleavage of a B–H bond, consistent with the character of the electronic state discussed in Section 3.2.1 and in line with the  observed for H_3_N–BH_3_. The second nonradiative pathway, characterized by the  in Figure 7, corresponds to the puckering of the pyridine ring. Interestingly, the puckering motion either involves the nitrogen atom (shown in the inset of Figure 7)—provoking an out-of-plane motion of the BH_3_ moiety–or the distortion of one or more carbon atoms of the ring. This nonradiative process closely resembles the nonradiative relaxation mechanism of isolated pyridine, discussed in detail in the literature.^74,75^ The concerted dissociation of two B–H bonds is not observed in the excited-state dynamics conducted for Py–BH_3_. However, we note that we cannot rule out a release of H_2_ upon relaxation of Py–BH_3_ to the ground state or following photoexcitation at higher energies.

Analysis of the wavelength dependence of the two deactivation channels reveals that the fraction of trajectories suffering a B–H dissociation or puckering is about the same for the first and second energy windows. However, the fraction of trajectories reaching the  increases substantially at higher excitation energies at the expense of the puckering relaxation process.

The substitution of NH_3_ with Py has dramatically changed the electronic structure and the underlying photochemistry of the B–N Lewis adducts. However, a B–N photodissociation pathway, yet again, remains elusive for this molecule at the excitation wavelength that we probed. Therefore, we propose an additional alteration of our model Lewis adduct by tuning its B–N bond strength. Weakening the strength of the B–N bond should, in principle, imply that the excited state with an n_N_ character decreases in energy. To achieve this goal, we used a weaker Lewis acid, boric acid B(OH)_3_, instead of the stronger BH_3_.

### 3.3 Photochemistry of Pyridine-Boric Acid

The last B–N model studied here is pyridine–boric acid, Py–B(OH)_3_. Py–B(OH)_3_ exhibits a longer B–N bond at its ground-state optimized geometry (1.73 Å) than that of H_3_N–BH_3_ (1.66 Å) and Py–BH_3_ (1.64 Å), in line with the expected weaker B–N bond for this molecule. To explore whether the weaker nature of the B–N bond in the ground electronic state has implications for the excited electronic properties of the molecule, we performed the same type of calculations as done for the other B–N adducts, starting by monitoring the electronic energies along a B–N relaxed scan.

#### 3.3.1 Photodissociation of the B–N Bond in Pyridine-Boric Acid

**Figure 8:**
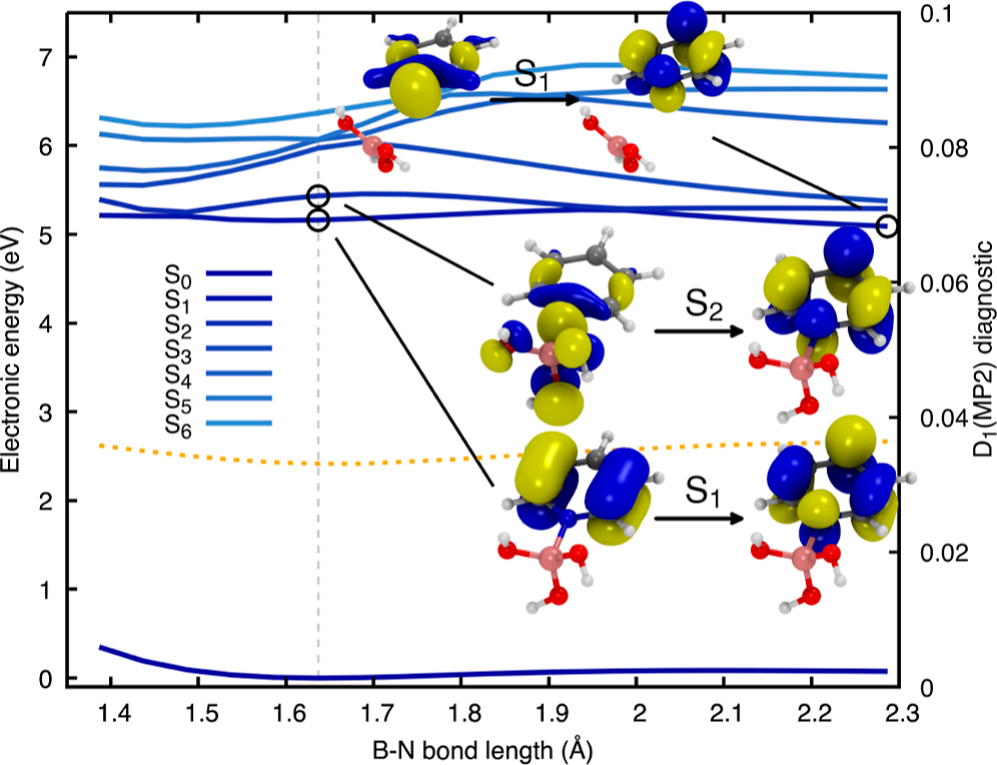
Relaxed scan along the B–N bond length of Py–B(OH)_3_ obtained for the ground electronic state with MP2/aug-cc-pVDZ. Excited electronic energies were obtained with ADC(2)/aug-cc-pVDZ. The FC point is indicated by the gray vertical dashed line. The lowest eight electronic states are calculated along the scan and depicted by lines in shades of blue–from S_0_ in dark blue to S_7_ in light blue. The D_1_ diagnostic for the MP2 ground state is reported as a dotted orange line. NTOs for the S_1_ and S_2_ excitations at the FC geometry are given as insets.

The relaxed scan obtained at the MP2 level of theory along the B–N bond of Py–B(OH)_3_ is shown in Figure 8. The variation of the ground-state electronic energy along the B–N relaxed scan highlights the weak nature of the B–N bond. The S_0_ electronic energy curve displays only a shallow minimum along this coordinate, in stark contrast with the minima observed earlier for H_3_N–BH_3_ and Py–BH_3_.

Focusing now on the excited electronic states at the FC geometry, we observe that the S_2_ electronic state exhibits an n_N_ → π* state (see NTOs, inset of Figure 8), again in line with a weaker B–N bond for this adduct. The S_2_ character at the FC geometry is similar to the n_N_ → π* states of Py–BH_3_, with an additional contribution coming from the lone pairs of the oxygen atoms on the boric acid moiety and a weaker contribution from the σ C–C and C–H bonds of the pyridine ring.

Following the n_N_ → π* character along the B–N relaxed scan confirms its stabilization upon a B–N bond elongation, in line with our earlier observation for Py–BH_3_. The diabatic crossing between the n_N_ → π* and the π → π* states occurs fairly close to the FC point (∼1.9 Å), in contrast with Py–BH_3_. This observation may imply that the n_N_ → π* state can be populated more easily in Py–B(OH)_3_ than in Py–BH_3_ during the nonadiabatic dynamics.

#### 3.3.2 Photoabsorption Cross-Section and Nonadiabatic Dynamics of Pyridine-Boric Acid

**Figure 9:**
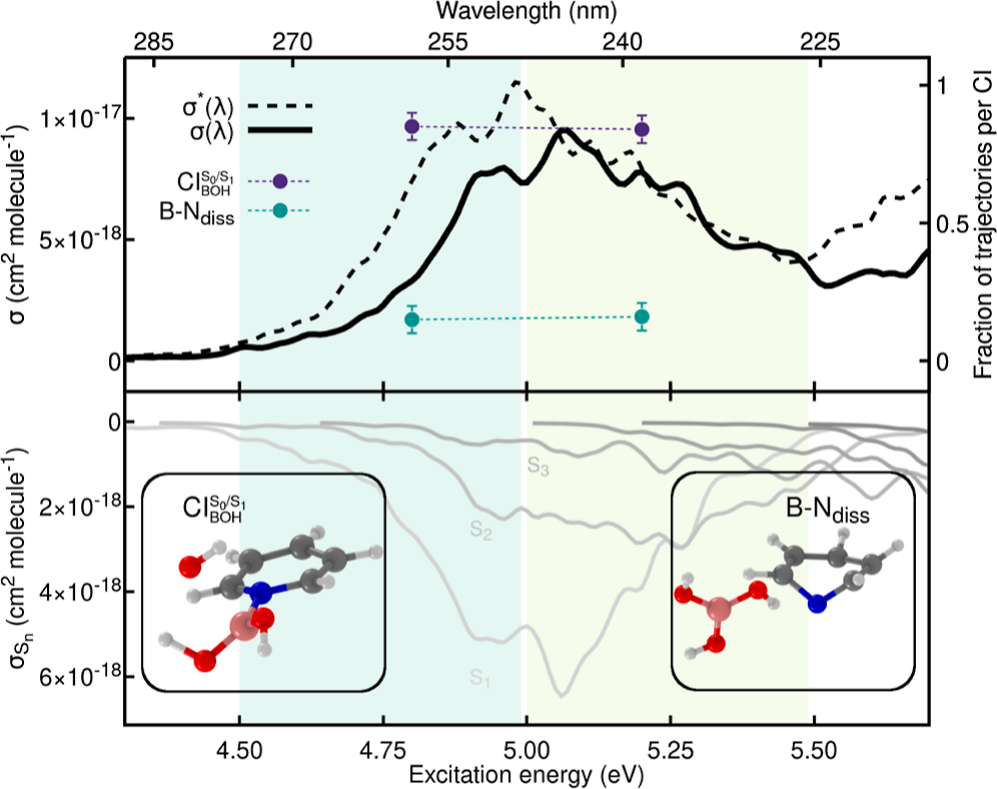
Photoabsorption cross-section and fractions of trajectories reaching a certain CI for Py–B(OH)_3_. Upper panel: full photoabsorption cross-section obtained with ADC(2)/cc-pVDZ (solid black line) and ADC(2)/aug-cc-pVDZ (dashed black line). The spectral range is partitioned into two energy windows shown as green and light green areas. The fraction of trajectories showing a B–OH dissociation (, purple) or a B–N dissociation (B–N_diss_, green) is determined for each energy window and reported with filled circles. Lower panel: individual excited-state contributions to the full photoabsorption cross-section depicted with solid lines colored from light gray (S_0_ → S_1_) to dark gray (S_0_ → S_8_). Exemplary molecular structures from the nonadiabatic molecular dynamics, representative of the CI decay for each of the two photodissociation pathways, are shown as insets.

We calculated the photoabsorption cross-section of Py–B(OH)_3_ and split it into two energy windows for the subsequent TSH nonadiabatic dynamics simulations (Figure 9). The first window aims to investigate the photochemistry of the molecules triggered by a photoexcitation in the S_1_ electronic state, with only minor contributions from excitation to the second excited electronic state S_2_. The higher excitation window is instead more complex as it incorporates multiple contributions from broad electronic-transition bands (see the lower panel of Figure 9). These broad energy range for the electronic transitions are linked to the weak B–N bond and, consequently, to the spread of the B–N distance in the sampled geometries used to calculate the photoabsorption cross-section (see Figure S3).

The nonadiabatic dynamics simulations reveal for both energy windows a nonradiative pathway involving a B–OH cleavage (purple circles in Figure 9)—analogous to the B–H dissociation observed in the nonadiabatic dynamics of Py–BH_3_. In addition, photoexcitation of Py–B(OH)_3_ resulted in direct B–N photodissociation (green circles in Figure 9). The B–N dissociation occurs in a few tens of femtoseconds and the B(OH)_3_ fragment is released in its ground electronic state. In contrast, the pyridine moiety is produced in an excited electronic state–in line with the results obtained in the relaxed scan presented above (Figure 8). The subsequent excited-state dynamics observed for pyridine is in line with earlier studies, where the molecule relaxes toward the ground state via a puckering motion. The puckering of pyridine occurring after the Py–B(OH)_3_ photodissociation should not be confused with the puckering mechanism observed in the excited-state dynamics of Py–BH_3_, as the B–N bond in the latter was still intact (see Figure 7). We note that the fast nonradiative decay of pyridine after the B–N photodissociation is expected to suppress any sizable fluorescence signal. Photodissociable Lewis adducts, decaying to the ground state via nonradiative mechanisms, have already been discussed in the literature in line with our findings.^16^ It is worth noting that the photodissociation of Py–B(OH)_3_ results in an excited Lewis base, but depending on the electronic states available for the Lewis base or acid, one may also expect that the photodissociation would result in an excited Lewis acid [pathway (e) in Figure 1]. Such a process was actually observed experimentally for conjugated Lewis acids, and fluorescence of both the adduct and the isolated excited Lewis acid were measured.^13,14,17,18^

### 3.4 Implications of the Theoretical Findings for the Interpretation of Earlier Photochemical Experiments on B–N Lewis Adducts

In the following, we propose to confront our newly gained understanding of the photophysics/photochemistry of B–N Lewis adducts with earlier experimental works. In particular, we will be looking to extract potential guiding principles for predicting the photochemistry of a given B–N Lewis adduct.

#### 3.4.1 B–R Photodissociation

An example of the photolysis of a Lewis adduct involving the rupture of the bond between the boron and its substituent is given by the photochemistry of tribenzylborane-ammonia adducts.^76^ Irradiating the tribenzylborane-ammonia complex in UV light led to an efficient and heterolytic photodissociation of the benzyl carbon–boron bond. This process mirrors the B–H photodissociation [pathway (b)] observed for H_3_N–BH_3_ and Py–BH_3_. The authors of this study also highlighted the formation of bibenzyl upon irradiation of the uncoordinated tribenzylborane, while only traces of this photoproduct were observed in the photochemistry of the tribenzylborane-ammonia adduct. The formation of bibenzyl could possibly be connected to the pathway (c) associated with the formation of H_2_ in the photochemistry of H_3_N–BH_3_, despite its small predicted quantum yield. Based on this comparison and our theoretical results, we may infer that a boron-based Lewis acid not embedded in a molecular framework might be prone to B–R bond breaking upon light irradiation.

#### 3.4.2 B–N Photodissociation

Shi et al. synthesized a series of molecules exhibiting frustrated, dynamic, and allowed B–N pairs. Among these molecules, two allowed B–N coordinated complexes display dative bond photodissociation.^10^ The B–N bond lengths of two of these complexes, one reversible and one allowed, are 1.805(2) and 1.792(3) Å, respectively. These bond lengths are comparable with the B–N distance in our Py–B(OH)_3_ adduct. Both of these complexes show photodissociation. However, the reversible complex also exhibits a thermodynamic cleavage of the B–N bond in the ground electronic state at modest temperatures (*T* > 298 K), while the allowed complex stays bounded for all temperatures under investigation (*T* < 371 K). These observations could be connected to our computational finding on Py–B(OH)_3_, which shows B–N photodissociation but is also prone to dissociate thermally due to the weak nature of its B–N bond. Furthermore, both the reversible and allowed Lewis adducts display only a weak emission in THF due to the presence of efficient nonradiative pathways. Yet, highly enhanced emission properties were observed in a THF/H_2_O solution due to the aggregation-induced emission effect and suppression of puckering and motions. This observation aligns with our findings that, upon B–N dissociation, one of the moieties forming the original Lewis adduct remains in an excited electronic state, and the availability of nonradiative decay pathways will dictate its emissive behavior (in the gas phase).

Matsuo et al., investigating the photochemistry of a planarized trinaphthylborane coordinated to pyridine, observed an unexpected dual fluorescence signal.^13^ The dual fluorescence was rationalized by the emission from (i) the excited trinaphthylborane–Py adduct and (ii) the excited trinaphthylborane formed following the excited-state photodissociation of the Lewis adduct. The latter process appears to follow pathway (e) highlighted in Figure 1 and contrasts with the sole pathway (a) observed for ammonia borane and Py–B(OH)_3_, where the Lewis base remained excited. The switch to pathway (e) for this molecule can be understood by the highly conjugated nature of the Lewis acid in trinaphthylborane. The findings by Matsuo and co-workers also suggest that, upon photoexcitation, the excited-state dissociation of the adduct was in competition with its decay via fluorescence (with a lifetime estimated to be 2.3 ns). One may therefore expect a rather slow excited-state dissociation process for the trinaphthylborane– Py, in comparison to the photodissociation observed in Py–B(OH)_3_, which takes place in a few hundreds of femtoseconds. This observation could be rationalized by two different effects. First, the trinaphthylborane– Py adduct appears to have a slightly stronger B–N bond (∼1.69 Å based on the X-ray structure obtained in ref (13) in line with the theoretical value of 1.69 Å obtained with MP2/cc-pVDZ) than Py–B(OH)_3_ (∼1.73 Å), which could make the excited-state photodissociation process less favorable. In addition, the photodissociation of the trinaphthylborane– Py adduct completely disappears at a lower temperature (*T* ∼ 193 K). Based on our theoretical analysis, the required B–N stretching necessary for the diabatic transition to occur and trigger the adduct photodissociation may not be possible at low kinetic energy or for too strong B–N bonds. Second, the trinaphthylborane– Py adduct does not possess efficient nonradiative decays, in contrast with H_3_N–BH_3_ and Py–BH_3_. This property allows the adduct to remain in the excited electronic state for long enough to either slowly photodissociate or decay via fluorescence. This slow process is not possible in H_3_N–BH_3_ and Py–BH_3_ due to the presence of the other deactivation channels [pathways (b)–(d)].

#### 3.4.3 Tuning the B–N Bond

Voegtle and Dawlaty investigated the strength of Lewis adducts created by combining BF_3_ to a series of quinoline derivatives, acting as photoactive Lewis bases.^77^ The focus of this work was not primarily on the photochemistry of the Lewis adducts but on how different quinoline-based Lewis bases could affect the strength of the B–N bond. A first observation from this work is that all quinolines showed an increased bonding affinity for BF_3_ when the adduct was in its first excited electronic state. This observation is consistent with the computational results obtained for our three models of Lewis adducts (in particular, ammonia borane, see Figure 2, for which the first excited electronic state is stabilized upon contraction of the B–N bond). The authors also highlighted a correlation between the Hammett parameter of the substituent groups and the ability of quinoline to coordinate more strongly with BF_3_ upon photoexcitation. This observation was linked to the change in electron density on the nitrogen atoms, being either depleted or enhanced by the functionalization with electron-withdrawing or electron-donating groups. A quinoline functionalized with an electron-donating group was a stronger Lewis photobase (i.e., binding more strongly BF_3_) than a quinoline functionalized with an electron-withdrawing group. This correlation resonates with our results, as an electron-rich acid (such as B(OH)_3_) acts as a weaker photoacid (i.e., binding less strongly a pyridine) than an electron-deficient acid (such as BH_3_). This can be qualitatively appreciated by comparing Figures 6 and 8. In the former, the S_1_ state is tangibly stabilized upon contraction of the B–N bond (i.e., BH_3_ acts as a moderate Lewis photoacid). In contrast, the S_1_ state remains shallow for the Py–B(OH)_3_ adduct, and only a minute stabilization is observed (i.e., B(OH)_3_ acts as a weak Lewis photoacid). We finally note that some B–N Lewis adducts may exhibit electronic states in which the B–N bond is weakened due to the decrease (increase) of the electron density on the nitrogen (boron).

## 4 Conclusions

In this work, we studied three different B–N Lewis adducts—H_3_N–BH_3_, Py–BH_3_, Py–B(OH)_3_—using a broad range of techniques in computational photochemistry. We used ammonia borane, H_3_N–BH_3_, as a model system to decipher the different nonradiative pathways of this class of molecules. This simple Lewis adduct exhibits a plethora of possible photochemical processes that can be accessed using different excitation wavelengths. In particular, we observed a B–H bond cleavage and a concerted bond breaking of two B–H bonds, combined with nonradiative decays triggered by N–H bond stretching. A similar B–H bond cleavage in the excited state was also observed for the Py–BH_3_ adduct, together with puckering distortions characteristic of the photochemistry of pyridine. Interestingly, the excited-state dynamics simulations for H_3_N–BH_3_ and Py–BH_3_ did not lead to a direct photodissociation of the B–N bond for the excitation wavelengths simulated. The adduct photodissociation was only observed for Py–B(OH)_3_, which presents a very weak B–N bond. The B–N photodissociation in Py–B(OH)_3_ competes with a phototriggered B–OH cleavage, in analogy with the B–H photodissociation mechanism of H_3_N–BH_3_ and Py–BH_3_.

Overall, our simulations describe many possible photochemical pathways for B–N Lewis adducts. In addition to the excitation wavelength, the importance of each pathway for a given B–N Lewis adduct will be dictated by the precise molecular nature of the Lewis acid and base and the strength of the adduct, as exemplified in our rationalization of the experimental results on these molecular systems available in the literature. If the Lewis acid forming the adduct possesses weak B–R bonds, they are likely to be readily photolyzed. In the absence of (i) a possible B–R bond dissociation and (ii) a nonradiative decay from the Lewis base or acid, B–N photodissociation could take place, further enhanced if the B–N bond is weak or there is sufficient internal energy to reach a dissociative character diabatically. The B–N photodissociation will lead to a photoexcited Lewis acid or Lewis base, depending on the stabilization of the excited electronic states of each moiety.

We believe that the possible photochemical routes highlighted in this work for B–N Lewis adducts will provide insights for the interpretation of upcoming photochemical experiments on this family of molecules and hopefully stimulate future photodynamics studies employing ultrafast spectroscopic techniques.

## Supporting Information Available

Detailed electronic-structure benchmark, raw results for the excited-state trajectories, active-space orbitals, and distribution of B–N bond lengths for the adducts studied in this work (PDF)
xyz geometries for the LIICs and scans presented in this work (ZIP)
