# Supplementary material for: A Theoretical Perspective on the Photochemistry of Boron–Nitrogen Lewis Adducts
Source: J Phys Chem A. 2024 Jan 18;128(6):996–1008. doi: 10.1021/acs.jpca.3c07016 (PMC10875676; doi:10.1021/acs.jpca.3c07016)
Supplement: Supplementary file 1 — jp3c07016_si_001.pdf [file jp3c07016_si_001.pdf]

Supporting Information: A Theoretical Perspective on the Photochemistry of Boron–Nitrogen Lewis Adducts

Emanuele Marsili and Basile F. E. Curchod*

Centre for Computational Chemistry, School of Chemistry, University of Bristol, Bristol BS8 1TS, UK

E-mail: basile.curchod@bristol.ac.uk

Supplementary Tables

Table S1: Summary of the outcomes of the TSH simulations for the three Lewis adducts investigated in this work.

	$H_3N{-}BH_3$		
window:	6 eV	7 eV	8 eV
$CI_{BH}^{S_0/S_1}$	13	17	13
$CI_{H_2}^{S_0/S_1}$	7	18	22
$CI_{NH}^{S_0/S_1}$	25	13	4
$N{-}B_{diss}$	0	0	0
discarded	5	8	13
total	50	56	52
	$Py{-}BH_3$		
window:	4 eV	5 eV	6 eV
$CI_{BH}^{S_0/S_1}$	10	12	29
$CI_{puckering}^{S_0/S_1}$	8	9	12
$N{-}B_{diss}$	0	0	0
discarded	9	9	21
total	27	30	52
	$Py{-}B(OH)_3$		
window:	4.75 eV	5.25 eV	
$CI_{BOH}^{S_0/S_1}$	50	38	
$N{-}B_{diss}$	9	7	
discarded	5	15	
total	64	60	

Table S2: Excitation energies in eV (rows with gray background) and oscillator strengths (rows with white background) of ammonia borane computed at the ground-state optimized geometry (SCS-MP2/def2-SVPD). The excited electronic states are labeled according to the C_{3v} point group.

	S_1/S_2 (E)	S_3/S_4 (E)	S_5 (A_2)	S_6 (A_1)	S_7/S_8 (E)	S_9 (A_1)
ADC(2)						
aug-cc-pVDZ	6.96	8.24	8.27	8.33	8.45	8.65
	(0.013)	(0.029)	(0.0)	(0.005)	(0.069)	(0.126)
aug-cc-pVTZ	7.07	8.34	8.39	8.42	8.55	8.82
	(0.014)	(0.036)	(0.0)	(0.005)	(0.062)	(0.129)
SCS-ADC(2)						
aug-cc-pVDZ	7.18	8.40	8.45	8.53	8.63	8.86
	(0.017)	(0.043)	(0.0)	(0.004)	(0.055)	(0.135)
aug-cc-pVTZ	7.31	8.52	8.59	8.64	8.75	9.04
	(0.017)	(0.051)	(0.0)	(0.004)	(0.048)	(0.138)
EOM-CCSD						
aug-cc-pVTZ	6.99	8.26	8.31	8.33	8.49	8.87
	(0.014)	(0.033)	(0.0)	(0.005)	(0.063)	(0.132)
aug-cc-pVQZ	7.02	8.31	8.35	8.39	8.51	8.91
	(0.014)	(0.033)	(0.0)	(0.005)	(0.060)	(0.130)
XMS(13)-CASPT2(8/9)						
aug-cc-pVDZ	6.81	8.14	8.16	8.20	8.40	8.71
	(0.010)	(0.004)	(0.0)	(0.0)	(0.060)	(0.140)

Table S3: Excitation energies in eV (rows with gray background) and oscillator strengths (rows with white background) of pyridine borane computed at the ground-state optimized geometry (MP2/aug-cc-pVDZ). The excited electronic states are labeled according to the C_s point group.

	S_1 (A")	S_2 (A")	S_3 (A')	S_4 (A")	S_5 (A')
ADC(2)					
cc-pVDZ	5.16	5.82	6.34	6.71	6.75
	(0.026)	(0.150)	(0.003)	(0.198)	(0.003)
aug-cc-pVDZ	5.07	5.62	6.15	6.44	6.58
	(0.031)	(0.156)	(0.003)	(0.202)	(0.004)
aug-cc-pVTZ	5.02	5.53	6.04	6.38	6.53
	(0.032)	(0.142)	(0.003)	(0.193)	(0.004)

Table S4: B−N bond lengths (Å) calculated at the optimized ground-state geometry for H_3N-BH_3, $Py-BH_3$, and $Py-B(OH)_3$ Lewis adducts (MP2/aug-cc-pVDZ).

	H_3N-BH_3	$Py-BH_3$	$Py-B(OH)_3$
B-N	1.66	1.64	1.73

Supplementary Figures

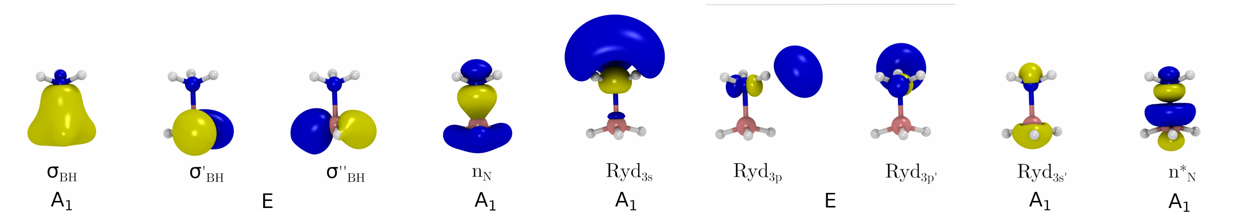

Figure S1: Representation of the SA(13)-CASSCF(8/9) natural orbitals and their labeling.

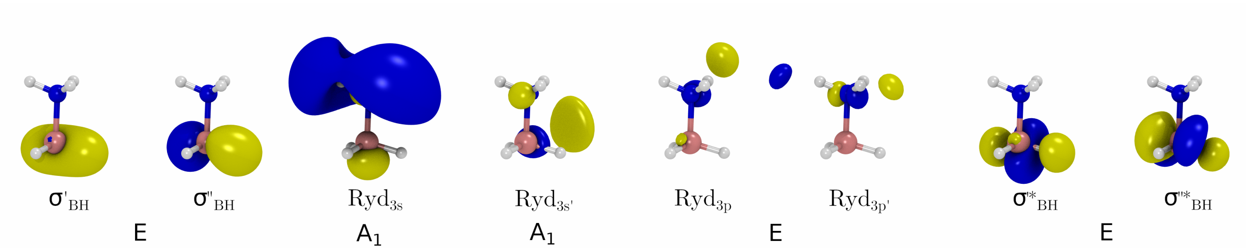

Figure S2: Representation of the SA(5)-CASSCF(4/8) natural orbitals and their labeling.

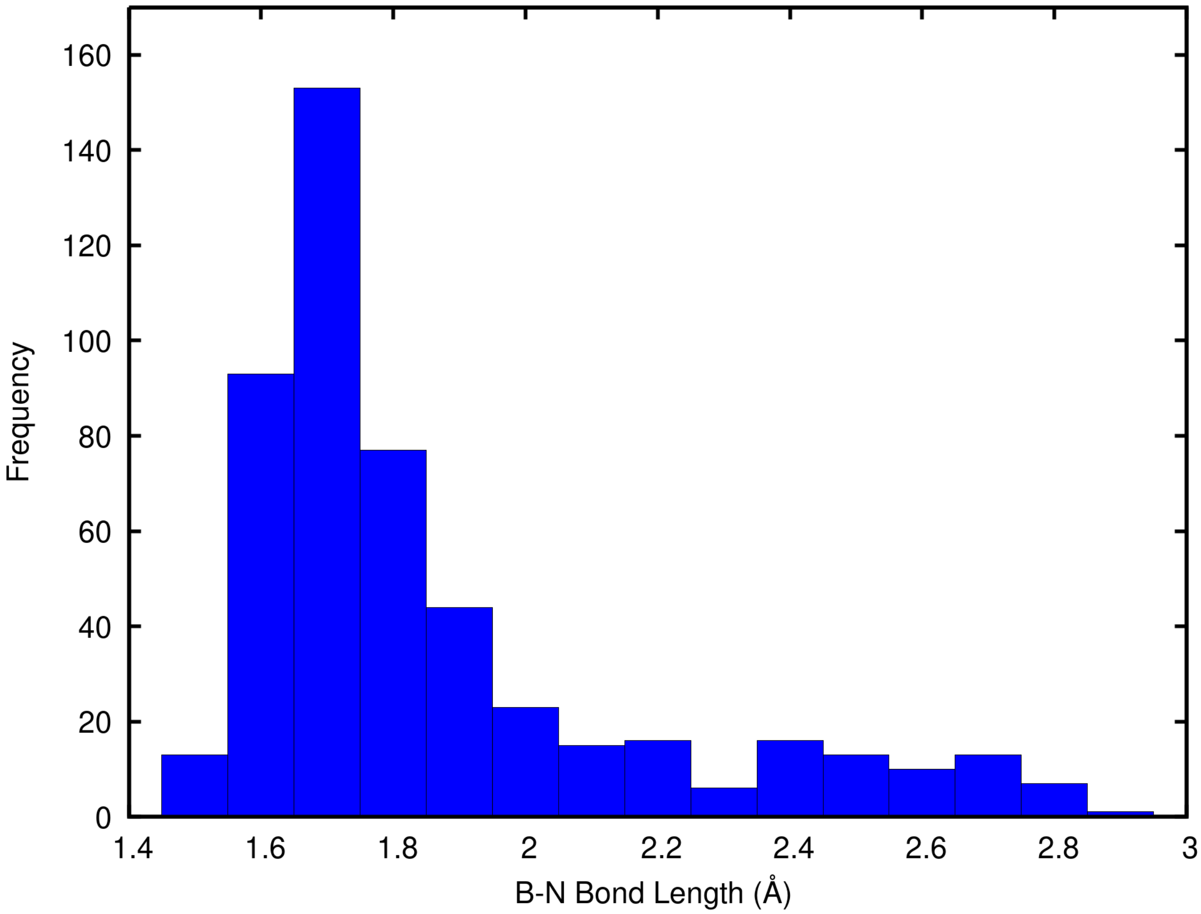

Figure S3: Distribution of the B−N distances for Py−B$(OH)_3$ collected during the QT ab initio molecular dynamics in the ground electronic state.

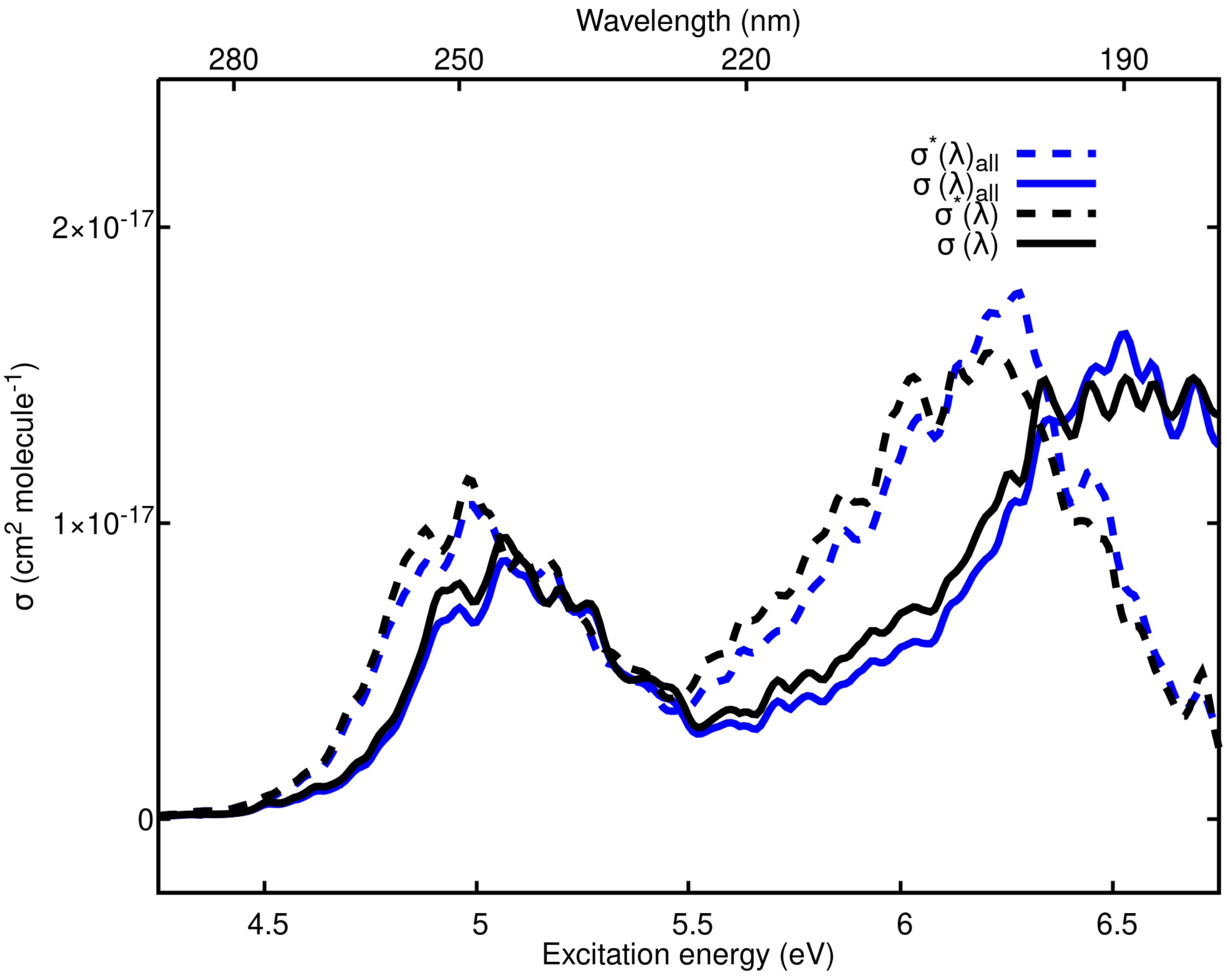

Figure S4: Photoabsorption cross-sections for Py−B(OH)$_3$ obtained (i) from the full set of 500 sampled geometries using ADC(2)/cc-pVDZ (dashed blue line) and ADC(2)/aug-cc-pVDZ (solid blue line) and (ii) from the subset of 403 sampled geometries exhibiting a B−N bond length shorter than 2.1 Å using ADC(2)/cc-pVDZ (dashed black line) and ADC(2)/aug-cc-pVDZ (solid black line).

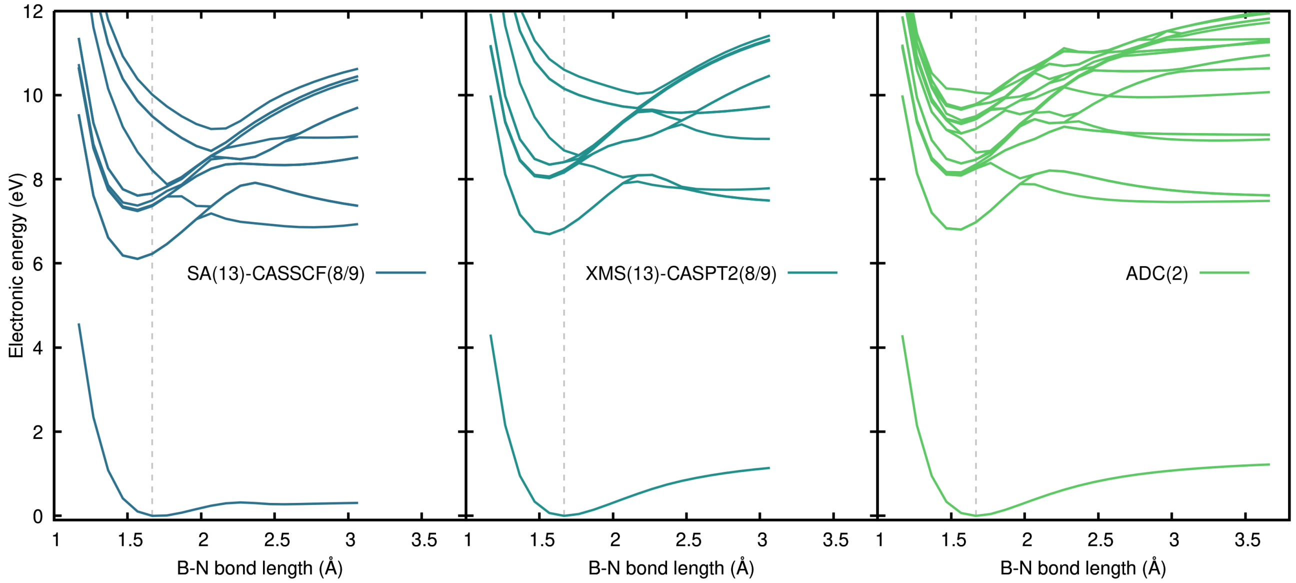

Figure S5: Electronic energies obtained for the ground-state relaxed scan (MP2/aug-cc-pVDZ) along the B−N bond length with SA(13)-CASSCF(8/9)/aug-cc-pVDZ (dark blue, left panel), XMS(13)-CASPT2(8/9)/aug-cc-pVDZ (blue-green, middle panel), and ADC(2)/aug-cc-pVDZ (green, right panel). The last points of the relaxed scan (long B−N bond lengths) are not reported for SA(13)-CASSCF(8/9)/aug-cc-pVDZ and XMS(13)-CASPT2(8/9)/aug-cc-pVDZ due to convergence issues. The location of the FC point (MP2) is indicated by a gray vertical dashed line.

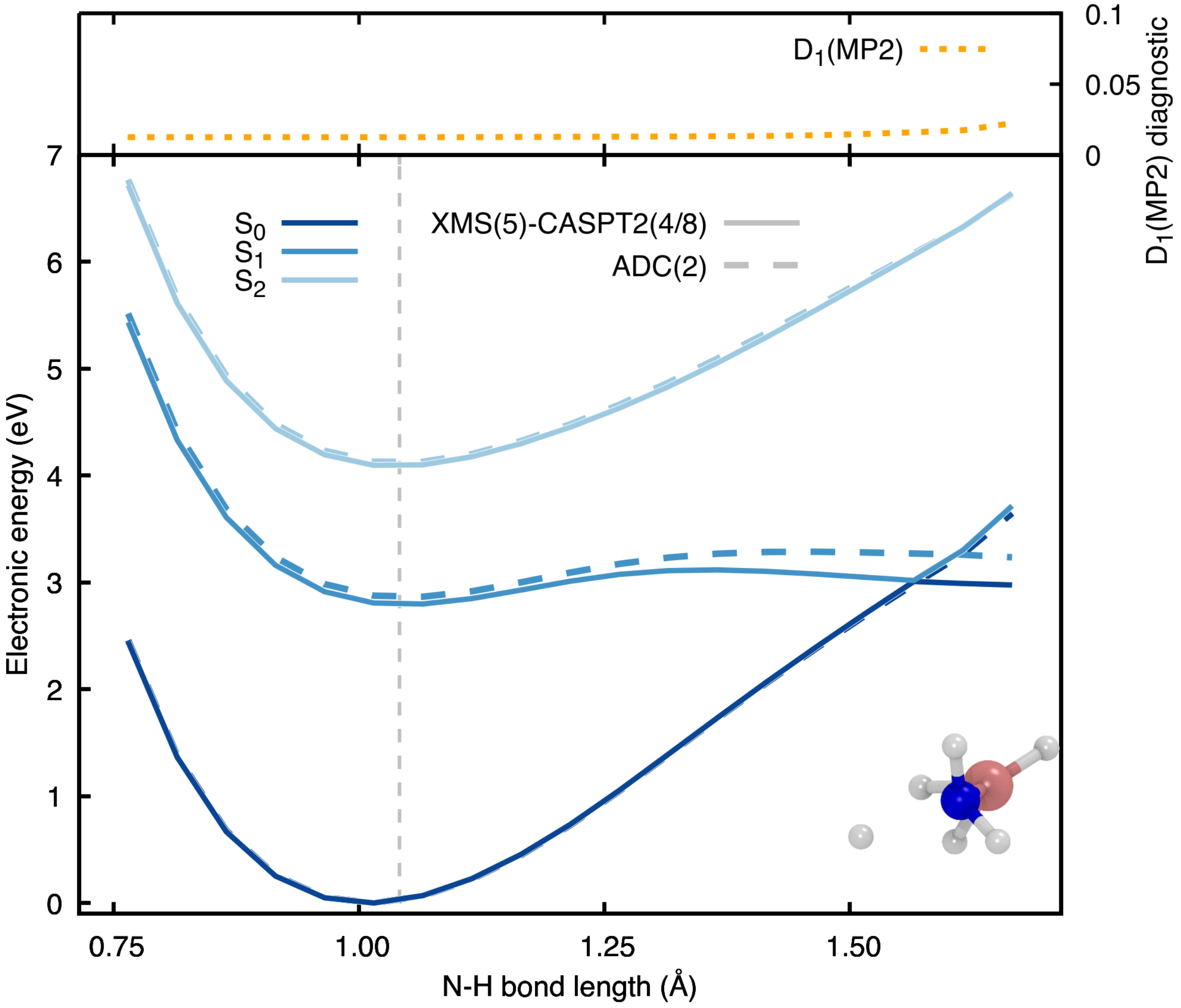

Figure S6: Relaxed scan along the N−H bond of H_3N-BH_3 for the the S_1 excited electronic state obtained with ADC(2)/aug-cc-pVDZ. The N−H bond length at the S_1 minimum-energy geometry is indicated by the gray vertical dashed line. The three lowest excited electronic states are depicted from dark (S_0) to light (S_2) blue, while the ADC(2)/aug-cc-pVDZ and XMS(5)-CASPT2(4/8)/aug-cc-pVDZ energies are indicated with dashed and solid lines, respectively. The D_1 diagnostic for the MP2 ground state is reported as a dotted orange line in the upper panel. The last structure of the relaxed scan is given as an inset.
